# Safety and efficacy of creatine supplementation in juvenile dermatomyositis: a randomized double-blind placebo-controlled cross-over trial

**DOI:** 10.1186/1546-0096-12-S1-P96

**Published:** 2014-09-17

**Authors:** Adriana Sallum, Marina Solis, Ana Paula Hayashi, Guilherme Artioli, Marcelo Sapienza, Maria Otaduy, Ana Pinto, Clóvis Silva, Rosa Pereira, Bruno Gualano

**Affiliations:** 1 School of Medicine, University of São Paulo, São Paulo, Brazil; 2 School of Physical Education and Sport , University of São Paulo, São Paulo, Brazil

## Introduction

Juvenile dermatomyositis patients may experience persistent weakness, loss of bone mass, and skeletal muscle atrophy as a long-term result of drug treatment and/or disease itself. In this regard, efforts to develop new therapeutic strategies able to attenuate these adverse outcomes have been considered of clinical relevance. It has been suggested that creatine supplementation could be safe, effective and inexpensive for treating idiopathic inflammatory myopathies, but no pediatric study has been conducted so far.

## Objectives

To examine the safety and efficacy of creatine supplementation in JDM patients

## Methods

JDM patients received placebo or creatine supplementation (0.1 g/kg/d) in a randomized, crossover, double-blind, repeated-measures design. Subjects were assessed at baseline and after 12 weeks, with 8-week washout period. Primary outcome was muscle function. Secondary outcomes included body composition, bone mineral density, biochemical markers of bone remodeling, inflammatory cytokines, aerobic conditioning, health-related quality of life, and disease-related parameters, dietary intake and muscle phosphorylcreatine (PCR) content. Safety was assessed by laboratory parameters and kidney function

## Results

Intramuscular PCR content was not significantly different between creatine and placebo before or after the intervention (Creatine - Pre: 21.4 ± 5.3, Post: 20.6 ± 2.7, delta score = -0.3 ± 2.5 mmol/kg wet muscle, ES = -0.15; Placebo - Pre: 20.4 ± 3.7, Post: 20.7 ± 3.6, delta score = -0.1 ± 4.2 mmol/kg wet muscle, ES = -0.15; 95% CI for delta score = -2.8 ± 2.4, p = 0.45 for interaction between creatine and placebo). No significant changes between placebo and creatine for muscle function and aerobic conditioning, body composition, bone mineral density, and quality of life were seen, probably due to the lack of change in intramuscular PCR content. Kidney function was not changed after creatine supplementation and no side effects were noticed.

## Conclusion

A 12-week creatine supplementation protocol is well tolerable and free of adverse effects but did not affect intramuscular PCR, muscle function, body composition, bone mineral density or quality of life in JDM patients.

## Disclosure of interest

None declared.

